# Dual-energy CT-based radiomics for predicting invasiveness of lung adenocarcinoma appearing as ground-glass nodules

**DOI:** 10.3389/fonc.2023.1208758

**Published:** 2023-08-10

**Authors:** Yuting Zheng, Xiaoyu Han, Xi Jia, Chengyu Ding, Kailu Zhang, Hanting Li, Xuexiang Cao, Xiaohui Zhang, Xin Zhang, Heshui Shi

**Affiliations:** ^1^ Department of Radiology, Union Hospital, Tongji Medical College, Huazhong University of Science and Technology, Wuhan, Hubei, China; ^2^ Hubei Province Key Laboratory of Molecular Imaging, Wuhan, China; ^3^ ShuKun (BeiJing) Technology Co., Ltd., Beijing, China; ^4^ Clinical Solution, Philips Healthcare, Shanghai, China

**Keywords:** dual-energy spectral computed tomography, radiomics, prediction, lung adenocarcinoma, ground-glass nodules, invasion

## Abstract

**Objectives:**

To explore the value of radiomics based on Dual-energy CT (DECT) for discriminating preinvasive or MIA from IA appearing as GGNs before surgery.

**Methods:**

The retrospective study included 92 patients with lung adenocarcinoma comprising 30 IA and 62 preinvasive-MIA, which were further divided into a training (n=64) and a test set (n=28). Clinical and radiographic features along with quantitative parameters were recorded. Radiomics features were derived from virtual monoenergetic images (VMI), including 50kev and 150kev images. Intraclass correlation coefficients (ICCs), Pearson’s correlation analysis and least absolute shrinkage and selection operator (LASSO) penalized logistic regression were conducted to eliminate unstable and redundant features. The performance of the models was evaluated by area under the curve (AUC) and the clinical utility was assessed using decision curve analysis (DCA).

**Results:**

The DECT-based radiomics model performed well with an AUC of 0.957 and 0.865 in the training and test set. The clinical-DECT model, comprising sex, age, tumor size, density, smoking, alcohol, effective atomic number, and normalized iodine concentration, had an AUC of 0.929 in the training and 0.719 in the test set. In addition, the radiomics model revealed a higher AUC value and a greater net benefit to patients than the clinical-DECT model.

**Conclusion:**

DECT-based radiomics features were valuable in predicting the invasiveness of GGNs, yielding a better predictive performance than the clinical-DECT model.

## Introduction

Lung cancer is the main cause of mortality among malignant tumors. Non-small cell lung cancer represents approximately 85% of all lung cancer cases, and adenocarcinoma is the primary histological type (1, 2). Ground-glass nodules (GGNs) refer to nodules with a slight increase in density on computed tomography (CT) that do not cover the bronchial structures or vascular edges (3). According to solid components, GGNs can be divided into pure GGNs (pGGNs) and part-solid nodules. When a GGN is persistent on HRCT, it usually indicates the existence of lung adenocarcinoma (LUAD) or its precursors, including invasive adenocarcinomas (IA), minimally invasive adenocarcinoma (MIA), adenocarcinoma *in situ* (AIS) and atypical adenomatous hyperplasia (AAH) (4–6), which can be difficult to distinguish without any additional investigation or more invasive measures.

Preinvasive-MIA and IA required different surgical approaches, postoperative treatments and had distinct prognoses. Limited wedge resection or segmental resection is typically performed for preinvasive-MIA to preserve maximum functional lung parenchyma, while lobectomy is always performed for IA to reduce tumor recurrence (7, 8). Thus, accurately predicting the invasiveness of lung adenocarcinoma before surgical decision-making is crucial for selecting appropriate surgical approach and improving prognosis.

Although some CT findings, such as maximal tumor diameter and density, have previously been shown to aid in identifying tumor invasion (9, 10), evaluating these characteristics relies on radiologists’ experience. Dual-energy CT (DECT) has emerged as a potential clinical diagnostic tool (11–13), enabling low-dose scanning, obtaining diagnostic images and various quantitative parameters unavailable with conventional CT. Recent studies (14, 15) have explored using DECT parameters, including CT60 keV values, virtual HU, and normalized iodine concentration (NIC), to assess the invasiveness of LUAD.

Radiomics can extract numerous quantitative features from medical images and convert them into minable data for subsequent analysis to support decision-making (16). Several studies showed the potential of radiomics to predict LUAD invasiveness based on conventional CT (17, 18). The developed radiomics models performed moderately well in predicting tumor invasion with areas under the curves (AUCs) of 0.77 and 0.79, respectively. However, to our knowledge, no previous radiomics analysis have examined the invasion of LUAD manifesting as GGNs based on DECT. It is of great interest whether DECT-based radiomics can improve the evaluation of tumor invasiveness.

Consequently, the aim of the study was to identify the value of DECT-based radiomics in differentiating preinvasive-MIA from IA characterized as GGNs and compared its predictive value with the clinical-DECT model.

## Materials and methods

### Patients

**Figure 1:**
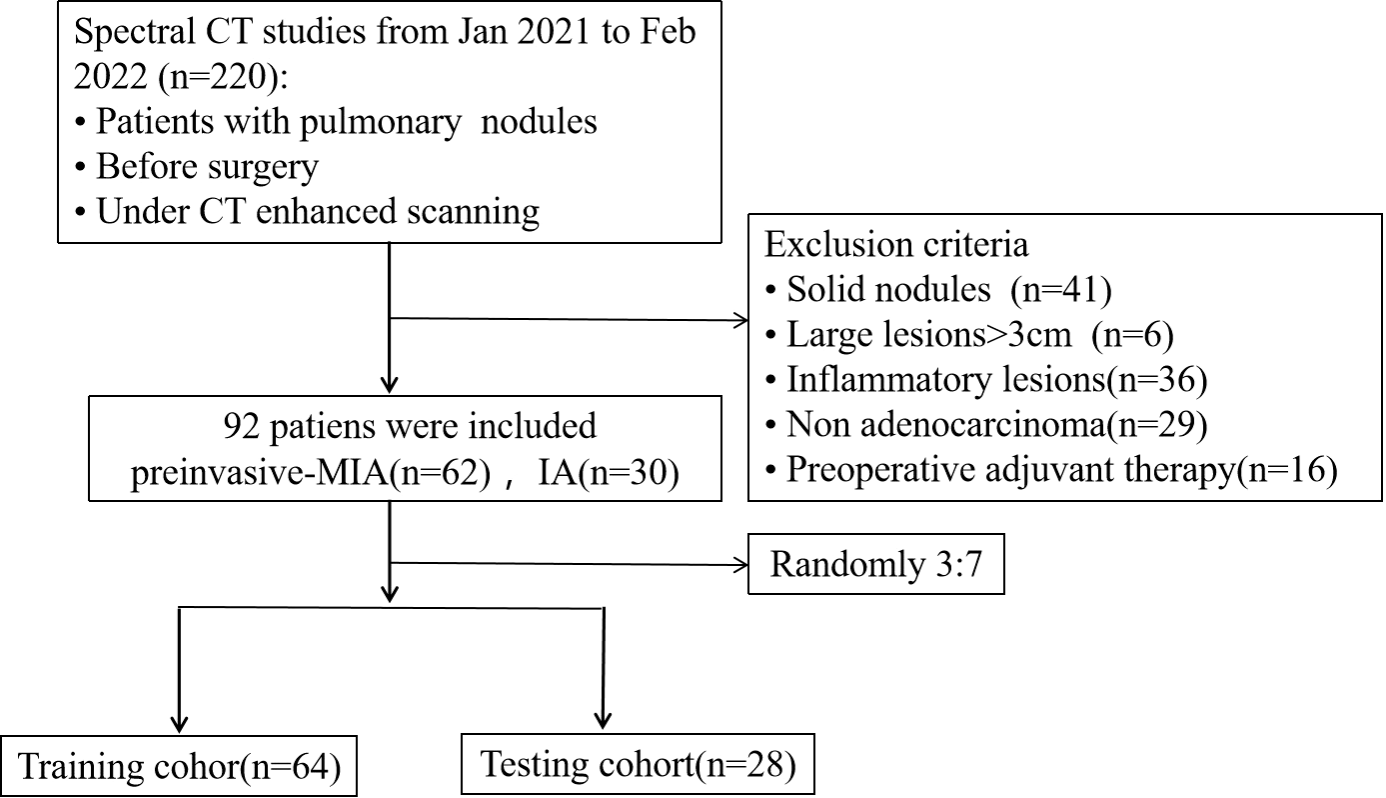
Study flowchart. MIA, minimally invasive adenocarcinoma; IA, invasive adenocarcinomas.

From January 2021 to February 2022, we included 220 patients with pulmonary nodules who performed DECT-enhanced scanning before surgery at our institution. 128 participants were removed due to (1): solid nodules (n=41) (2); large lesions over 3cm (n=6) (3); inflammatory lesions (n=36) (4); non-adenocarcinoma (n=29) (5); preoperative adjuvant therapy (n=16). Thus, 92 patients were included with 62 cases of preinvasive-MIA and 30 cases of IA and then were randomly assigned to a training (64 patients) and test cohort (28 patients), with a ratio of 7:3 (**Figure 1**).

### Pathological analysis

All surgical specimens were fixed in formalin and stained with hematoxylin-eosin as routinely prescribed by our hospital. Two pathologists examined the specimens and recorded the pathological subtype of each tumor following the International Association for the Study of Lung Cancer (IASLC)/American Thoracic Society (ATS)/European Respiratory Society (ERS) (19). All GGNs were separated into preinvasive-MIA (AAH, AIS and MIA) and IA groups.

### DECT acquisition

Following the standard procedure, the CT scans were taken using a dual-layer spectral CT (IQon; Philips Healthcare). The acquisition parameters were as follows:120kVp, with the automatic regulation of the tube current; 512 × 512 matrix; collimation 64 × 0.625 mm; reconstructed slice thickness and interval 1.5 mm/1.5 mm. A contrast medium (Iohexol, 320 mg/ml) was injected intravenously at a rate of 2–2.5 ml/s. Conventional images were reconstructed using the iDose4 (Philips Healthcare) algorithm, and spectral-based images (SBIs) were reconstructed using a spectral reconstruction algorithm.

### Image analysis

Two radiologists (HSS and YTZ, with 31 and 2 years of experience in thoracic radiology, respectively) blind to the clinical and histologic findings analyzed CT images and measured the quantitative parameters in the artery phase (AP) and venous phase (VP). Tumor size and density (part-solid nodules and pGGNs) were chosen to help identify tumor invasion in patients with LUAD.

Spectral CT images were quantitatively analyzed by commercially available tools (IntelliSpace Portal v. 10.1, Philips Healthcare). Reconstructed images were as follows: iodine-based material decomposition images, effective atomic number (Zeff) images, and 101 sets of virtual monochromatic images (VMIs). The rules for measuring GGNs were shown below (20, 21): (I) Select a region-of-interest (ROI) to cover over 70% of the tumor area in the largest slice, avoiding major vessels and bubbles. (II) Keep the ROIs consistent in size and position across different image types and phases. (III) Take the average values of 2 independent measurement results for analysis and comparison. The following imaging parameters were acquired: CT attenuation values of nodules from VMIs (including 40 kev and 100 kev), iodine concentration (IC), and Zeff at AP and VP. Since the body weight, circulation status and body composition could affect IC values, they were standardized to that of the aorta in the same slice to obtain the normalized iodine concentration (NIC): NIC = IC _Ca_/IC _aorta_, as previously described (22). Mean IC values were recorded for lesions and aorta ROIs. The slope of the spectral curve [slope(k)] was computed: slope(k) = |CT number (40 keV) − CT number (100 keV) |/60.

### DECT images segmentation

**Figure 2:**
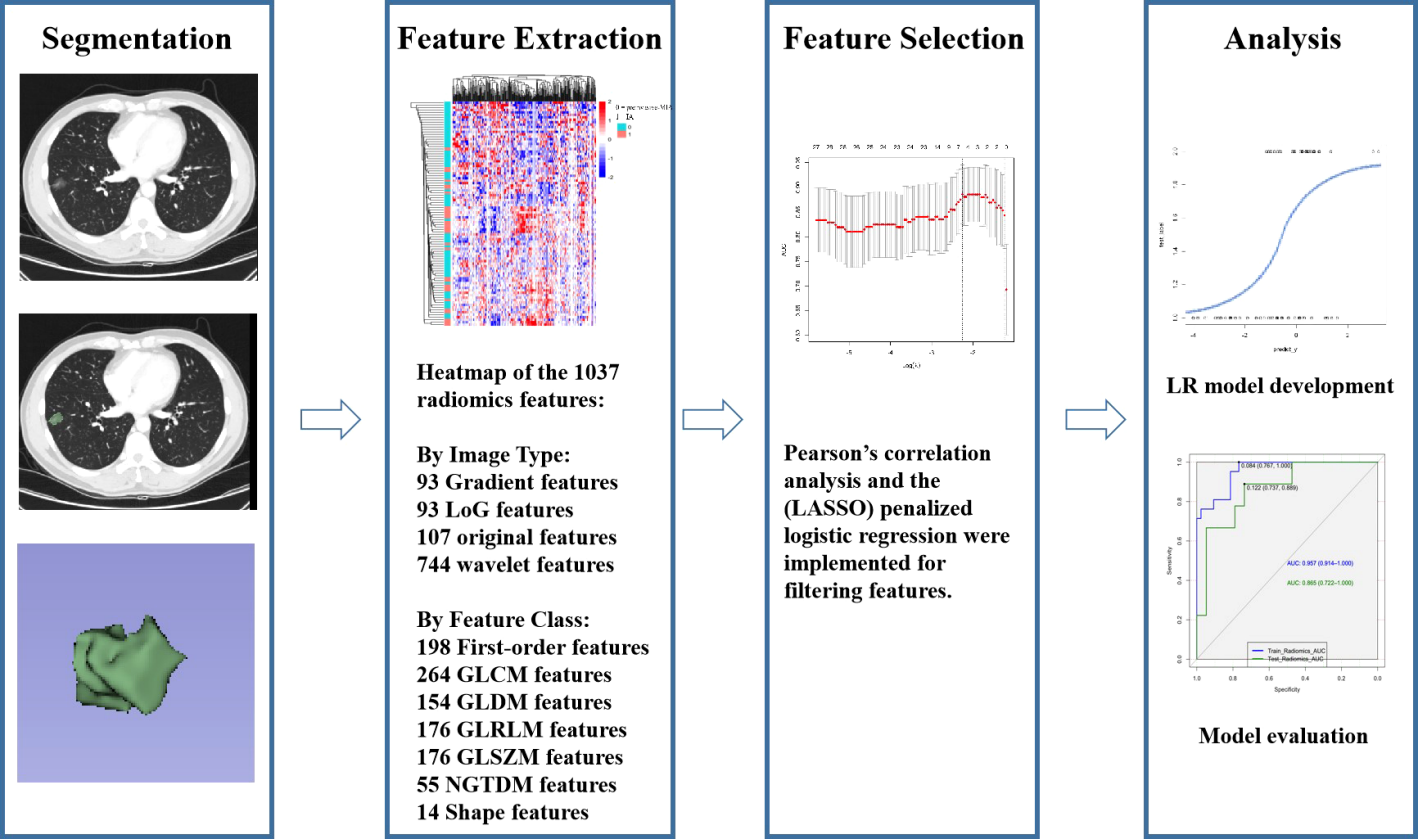
Radiomics workflow.

Studies (23, 24) have shown that VMIs generated by spectral CT can improve reproducibility in measuring part-solid nodules. The monochromatic reconstructions at 50-55 keV yield the best image quality for lung parenchyma. Thus, we chose to delineate tumors on 50kev monoenergetic images. Two junior radiologists (XH and YZ with 6 and 2 years of experience in thoracic imaging, respectively) delineated the ROI on DECT images semi-automatically layer by layer using 3D-slicer software. The 3D-slicer software was then used to automatically reconstruct the three-dimensional volumes of interest (3D-VOIs) (**Figure 2**). To evaluate intra-and inter-observer repeatability of feature extraction, images from 30 patients were randomly chosen and independently segmented on the target slice by the same radiologist (YZ) after one week and another radiologist (SHS) with 31 years of experience, respectively. Intra-and inter-observer agreement in the imaging evaluation was assessed by the intraclass correlation coefficient (ICC) (25).

### Radiomics feature extraction

Studies (26, 27) have shown that venous phase images can better reflect tumor microcirculation and result in high clinical usefulness. Low-energy images (40–70 keV) in VMI can substantially increase vascular contrast, while high-energy images(80-190kev) can decrease the metallic artifact to improve the detection of lung nodules (28). As a result, the data in venous phase were transmitted to workstations to generate VMIs with 50 keV and 150 keV energy levels for radiomics investigation. A Radiomics system (ultra scholar, ShuKun (BeiJing) Technology) was used for the extraction of radiomics features, and a third-party Python library called Pyradiomics was used for extracting all the radiomics features in the feature extraction module (29). Before radiomics examination, each 3D CT image was resampled to a spacing of (0.7, 0.7, 1.5) mm using a B-spline curve interpolation algorithm. 1037 3D-radiomics features were extracted for each VOI (types and numbers are displayed in **Figure 2**, and details are available at pyradiomics.readthedocs.io/en/latest/features.html).

### Delta- and mean-radiomics features

For DECT scans with 50 keV and 150 keV energy levels, radiomics features (RFs) were extracted for these two energy levels, respectively. The delta-RFs referred to the relative net change of RFs between two energy levels (30):


Relative Net Change = (Feature150kev – Feature50kev)/Feature50kev

And mean-RFs referred to the mean values of RFs between two energy levels:


Mean RF = (Feature150kev + Feature50kev)/2

### Statistical analysis

All statistical analyses were carried out using R (version 4.0.2; http://www.Rproject.org) and the SPSS software (SPSS, version 21, IBM, Chicago, IL, USA). The ‘glmnet’ package was used for LASSO binary logistic regression. The ‘rms’ package was used for multivariate binary logistic regression. The ‘pROC’ program was employed to conduct receiver operating characteristic (ROC) analysis. Categorical variables were represented as frequency (percentage) and Fisher’s exact X^2^ test was applied to compare categorical variables. We used the Shapiro-Wilk test to check the normality assumption of the continuous data. When the data were normally distributed, they were expressed as mean ± standard deviation and the independent sample t test was applied to compare them. If the data distribution was not normal, they were represented as median (interquartile range, IQR) and the Mann-Whitney U test was employed for the nonparametric analysis. P<0.05 (two-tailed) was regarded as statistically significant.

All radiomics features were normalized to the z-score. To eliminate redundant and unstable features, ICC calculation and Pearson’s correlation analysis were conducted (r>0.8, ICC>0.75). Least absolute shrinkage and selection operator (LASSO) analysis was carried out to identify characteristics for further evaluation (31). The maximum area under the curve (AUC) and five-fold cross-validation served as features filtering criteria. A multivariate logistic regression (LR) algorithm established a classification model based on the selected features. Two models were then constructed: a clinical-DECT model incorporating clinical and DECT quantitative parameters and a radiomics model based on the radiomics features. The accuracy, sensitivity and specificity were calculated for each model. The confounder matrix illustrated the predictive ability. The DeLong test was performed to verify the efficiency of different models for diagnosis. Moreover, we conducted a decision curve analysis (DCA) to evaluate the clinical value of the models by estimating the net benefits under different threshold probabilities.

## Results

### Clinical characteristics and quantitative DECT results of adenocarcinoma

**Figure 3:**
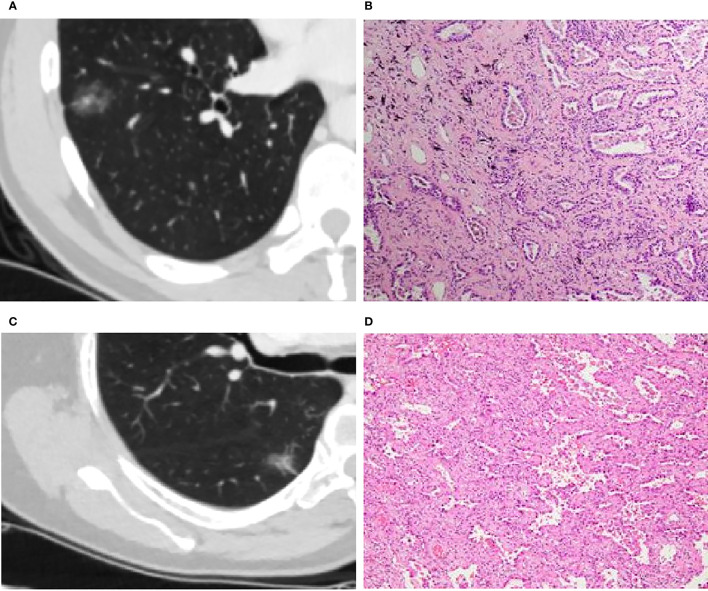
**(A, B)**: Invasive adenocarcinoma (IA) in a 44-year-old man with a part-solid nodule **(A)**. Photomicrograph (H&E staining, ×100) confirmed IA **(B)**. **(C, D)**: Minimal invasive adenocarcinoma (MIA) in a 66-year-old woman with a part-solid nodule **(C)**. Photomicrograph (H&E staining, ×100) confirmed MIA **(D)**.

The study included 62 preinvasive-MIA and 30 IA patients. **Table 1** summarizes the clinical characteristics, main CT features (tumor size and tumor density type) and quantitative results (IC, Zeff and slope[k]) of lesions. Significant differences could be found in age, sex, smoking and alcohol history (all *p <0.05*). Considering CT features, IA was typically larger than preinvasive-MIA (15.5 mm *vs.* 10 *mm*, *p* < 0.001). Tumor density differed between the IA and preinvasive-MIA group (*p* < 0.001). Most IA manifested as part-solid nodules (25/30, 83.3%; **Figure 3**). Regarding quantitative parameters, there was significant difference in Zeff at AP and VP (*p =* 0.008, *p =* 0.003, respectively). NIC was larger in IA than in preinvasive-MIA at VP (*p =* 0.04). However, no significant difference was found in slope (k) values between the groups.

**Table 1:** Comparison of clinical characteristics and DECT parameters of GGNs between preinvasive-MIA and IA.

Variable	Preinvasive-MIA (n=62)	IA (n=30)	P value
Age (years)	50.4 ± 13.3	59.4 ± 8.6	<0.001*
Sex			0.009*
Male	9 (14.5%)	12 (40%)	
Female	53 (85.5%)	18 (60%)	
Smoking history			0.005*
No	60 (96.8%)	23 (76.7%)	
Yes	2 (3.2%)	7 (23.3%)	
Alcohol history			0.037*
No	61 (98.4%)	26 (86.7%)	
Yes	1 (1.6%)	4 (13.3%)	
Density			<0.001*
pGGN	35 (56.5%)	5 (16.7%)	
Part-solid nodule	27 (43.5%)	25 (83.3%)	
Maximum diameter (mm)	10 (8,13)	15.5 (13,21.5)	<0.001*
AP			
Zeff	8.72 (8.16,9.02)	8.5 (8.14,9.0)	0.008*
Spectrum curve slope	1.42 (0.9,1.82)	1.61 (1.07,2.17)	0.339
NIC	0.14 (0.08,0.21)	0.14 (0.11,0.22)	0.281
VP			
Zeff	8.51 (8.21,8.93)	8.27 (7.89,8.46)	0.003*
Spectrum curve slope	1.3 ± 0.6	1.5 ± 0.8	0.136
NIC	0.28 (0.19,0.36)	0.33 (0.25,0.44)	0.04*

*P< 0.05 based on comparisons between the two groups. Data are median (IQR) or n/N (%). MIA, minimally invasive adenocarcinoma; IA, invasive adenocarcinomas; pGGN, pure-ground opacity nodule; AP, artery phase; VP, venous phase; Zeff, effective atomic number; NIC, normalized iodine concentration.

**Figure 4:**
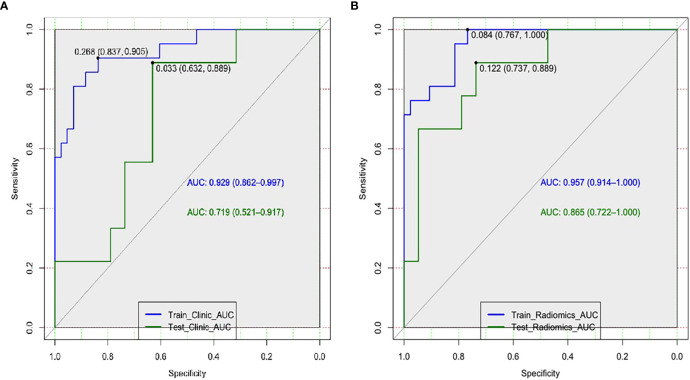
Receiver operating characteristic curves of the clinical-DECT model **(A)** and the radiomics model **(B)** in the training and test sets.

Afterwards, the clinical-DECT model was established by LR algorithm (**Table 2**), including sex, age, smoking, alcohol history, tumor size, density, Zeff, and NIC, with an AUC of 0.929 (sensitivity of 90.5% and specificity of 69.8%) in the training cohort and an AUC of 0.719 (sensitivity of 55.6% and specificity of 73.7%) in the test cohort (**Figure 4A**). **Table 3** illustrated the distribution of selected clinical and DECT characteristics in the training and test set.

**Table 2:** Features included in clinical-DECT model and their coefficients.

	Estimate	Std.Error	Z value	Pr (>|z|)
(Intercept)	19	17.6	1.08	0.28
Age	0.056	0.054	1.02	0.31
Sex	1.82	1.23	1.48	0.14
Smoking history	15.06	2399.55	0.01	0.99
Alcohol history	-13.35	2399.55	-0.01	1
Tumor size	0.32	0.14	2.35	0.02
Density	0.33	1.15	0.29	0.78
AP_ Zeff	-0.24	0.6	-0.4	0.69
VP_ Zeff	-3.5	2.28	-1.54	0.12
VP_ NIC	-1.59	8.27	-0.19	0.85

AP, artery phase; VP, venous phase; Zeff, effective atomic number; NIC, normalized iodine concentration.

**Table 3:** Comparison of selected clinical characteristics and DECT parameters of GGNs between preinvasive-MIA and IA in the training and test cohorts.

	Train cohort			Test cohort		
Variable	Preinvasive-MIA (n=43)	IA (n=21)	P value	Preinvasive-MIA (n=19)	IA (n=9)	P value
Age (years)	53.3 ± 12.9	59.6 ± 9.2	0.051	43.8 ± 12.2	58.9 ± 7.4	0.002*
Sex			0.02*			1
Male	5 (11.6%)	10 (47.6%)		4 (21.1%)	2 (22.2%)	
Female	38 (88.4%)	11 (52.4%)		15 (78.9%)	7 (77.8%)	
Smoking history			0.003*			0.574
No	43 (100%)	16 (76.2%)		17 (89.5%)	7 (77.8%)	
Yes	0 (0%)	5 (23.8%)		2 (10.5%)	2 (22.2%)	
Alcohol history			0.032			1
No	43 (100%)	18 (85.7%)		18 (94.7%)	8 (88.9%)	
Yes	0 (0%)	3 (14.3%)		1 (5.3%)	1 (11.1)	
Density			0.007*			0.039*
pGGN	24 (55.8%)	4 (19%)		11 (57.9%)	1 (11.1%)	
Part-solid nodule	19 (44.2%)	17 (81%)		8 (42.1%)	8 (88.9%)	
Maximum diameter (mm)	11.4 (9,14)	15 (12.5,19.5)	<0.001*	10 (8,12)	18 (14.5,26.5)	<0.001*
AP_ Zeff	8.74 (8.08,9.17)	8.54 (8.14,9.0)	0.04*	8.65 (8.31,8.89)	8.24 (8.02,8.56)	0.066
VP_ Zeff	8.54 (8.22,8.97)	8.12 (7.88,8.39)	0.006*	8.47 ± 0.36	8.32 ± 0.29	0.281
VP_ NIC	0.25 (0.15,0.34)	0.31 (0.22,0.40)	0.281	0.28 ± 0.14	0.37 ± 0.1	0.104

*P< 0.05 based on comparisons between the two groups. Data are median (IQR) or n/N (%). MIA, minimally invasive adenocarcinoma; IA, invasive adenocarcinomas; pGGN, pure-ground opacity nodule; AP, artery phase; VP, venous phase; Zeff, effective atomic number; NIC, normalized iodine concentration.

### Radiomics model building and validation

**Figure 5:**
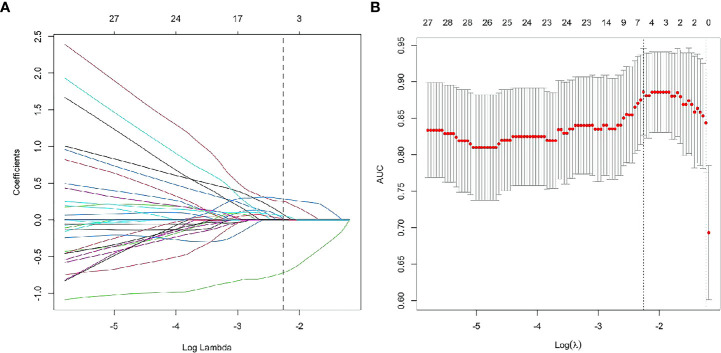
Radiomics feature selection using least absolute shrinkage and selection operator (LASSO) logistic regression. LASSO logistic regression of radiomics features **(A)** and the AUC versus the regularization parameter lambda **(B)**.

According to Pearson’s correlation analysis results, 332 delta-radiomics features were selected to build the delta-radiomics model. Then, 138 features were preserved from 50kev images by ICC and PCC. Based on the mean-RFs mentioned above, 276 features were derived combined with the features of 150kev images. According to LASSO penalized logistic regression analysis, seven features (three first order and four second order parameters, including GLCM and GLRLM features) revealed a significant correlation between radiomics and adenocarcinoma invasiveness (**Figures 5A, B**). Then, these radiomics features were taken into the LR model to establish a radiomics model. **Table 4** illustrated the features included in the radiomics model and their coefficients. The radiomics model performed well in the training (AUC: 0.957, sensitivity: 95.2%, and specificity: 79.1%) and test cohorts (AUC:0.865; sensitivity:88.9%, and specificity: 73.7%) (**Figure 4B**).

**Table 4:** Features included in radiomics model and their coefficients.

	Estimate	Std. Error	z value		Pr (>|z|)
(Intercept)	-1.54	0.71	-2.15		0.03
delta_wavelet.LLH_firstorder_Median	1.99	3.14	0.63		0.52
mean_wavelet.LHL_glrlm_LongRunEmphasis	0.64	0.77	0.83		0.40
mean_wavelet.HLL_glrlm_LongRunHighGrayLevelEmphasis	0.94	0.69	1.36		0.17
mean_wavelet.LLL_firstorder_RobustMeanAbsoluteDeviation	1.42	0.76	1.86		0.06
mean_log.sigma.6.0.mm.3D_firstorder_10Percentile	-1.65	1.04	-1.57		0.11
mean_log.sigma.6.0.mm.3D_firstorder_InterquartileRange	-0.87	0.79	-1.10		0.27
mean_log.sigma.6.0.mm.3D_glcm_ClusterShade	0.10	0.50	0.20		0.84

The rad-score of each lesion was computed according to the following formula:


$$\begin{array}{l} \text{Rad-Score }=\text{ -}1.54+1.99*\text{delta\_wavelet.LLH\_firstorder\_Median}\\ \;+0.64*\text{mean\_wavelet.LHL\_glrlm\_LongRunEmphasis}\\ \;+0.94*\text{mean\_wavelet.HLL\_glrlm\_LongRunHighGrayLevelEmphasis}\\ \;+1.42*\text{mean\_wavelet.LLL\_firstorder\_RobustMeanAbsoluteDeviation}\\ \;\text{ -}1.65*\text{mean\_log.sigma}.6.0.\text{mm}.3\text{D\_firstorder\_}10\text{Percentile}\\ \text{ -}0.87*\text{mean\_log.sigma}.6.0.\text{mm}.3\text{D\_firstorder\_InterquartileRange}\\ \;+0.10*\text{mean\_log.sigma}.6.0.\text{mm}.3\text{D\_glcm\_ClusterShade} \end{array}$$

**Figure S1** displayed the rad scores significantly higher in IA than in preinvasive-MIA (p <0.001) in the training and test cohorts.

### Performance comparison between different models

In comparison with the clinical-DECT model, the radiomics model exhibited improved predictive ability in both the training and test cohorts (AUC, 0.957 *vs* 0.929, *p =* 0.341; 0.865 *vs* 0.719, *p =* 0.09, respectively). **Table 5** summarized the findings of confounder matrix analysis. The radiomics model exhibited better accuracy, sensitivity, and specificity than the clinical-DECT model in the training and test cohorts.

**Table 5:** Confounder matrix for the training and test sets in the two models.

Predicted results	Actual results		Accuracy (%)	Sensitivity (%)	Specificity (%)
	Preinvasive-MIA	IA			
Clinical-DECT model					
**Training data set**			76.6	90.5	69.8
Preinvasive-MIA	30	2			
IA	13	19			
**Testing data set**			67.9	55.6	73.7
Preinvasive-MIA	14	4			
IA	5	5			
Radiomics model					
**Training data set**			84.4	95.2	79.1
Preinvasive-MIA	34	1			
IA	9	20			
**Testing data set**			78.6	88.9	73.7
Preinvasive-MIA	14	1			
IA	5	8			

Rows correspond to the prediction of the logistic algorithm, and columns to known outcomes. MIA, minimally invasive adenocarcinoma; IA, invasive adenocarcinomas.

### Clinical use

DCA assessed the clinical utility of the two predictive models (**Figure S2**). Compared to the treat-all and treat-none models, both the clinical-DECT and radiomics models provided a net benefit to patients, with the radiomics model showing superior benefits. **Figure S3** showed waterfall plots of the rad-score for the test and training sets, indicating that the model can effectively distinguish IA from preinvasive-MIA.

## Discussion

The aim of the study was to investigate the predictive value of DECT-based radiomics for identifying the invasiveness of GGNs. We evaluated the main radiographic features, measured quantitative parameters, and extracted radiomics features from VMI (50kev and 150kev images). Our findings confirmed that the DECT-based radiomics model was superior to the clinical-DECT model in distinguishing IA from preinvasive-MIA, providing a simple and reliable prediction method for accurately determining tumor invasiveness before surgery.

As expected, our study found that IA tended to be larger and part-solid nodules on CT images, similar to the findings of previous studies (9, 32). For quantitative parameters from DECT in our study, Zeff and NIC were considered significant differentiators of IA from preinvasive-MIA. Zeff represents the composite atom, which can be used to identify material composition (12). Li et al. (33) found that tumors with a predominant pattern of solid/micropapillary had lower Zeff than those with a predominant pattern of lepidic/acinar/papillary. Consistent with the previous study, the Zeff of IA was smaller than that of preinvasive-MIA in our research. This finding may be because preinvasive-MIA tends to have lepidic growth patterns, whereas IA can have the more aggressive adenocarcinoma subtype (solid and micropapillary patterns) (34). The result confirmed the value of Zeff in differentiating IA from preinvasive-MIA. In contrast to the finding of Zhang et al. (35), the NIC in IA was higher than in preinvasive-MIA in our study, suggesting that IA had more underlying microvascular and tumor angiogenesis. The observation can be explained as we included the part-solid nodules, and carefully avoided vessels when outlining the ROIs. In addition, we observed no statistical difference in slope(k) at both VP and AP, consistent with Zhang et al. (35). It indicated that different histological subtypes of adenocarcinomas manifested as GGNs had similar changes in iodine concentration.

Radiomics analysis could use the information from VMI datasets to expand new horizons for noninvasively assessing tumor signatures. One study (36) found that the radiomics model based on 70keV images showed good performance in predicting head and neck squamous carcinoma differentiation. Another study (37) demonstrated that radiomics derived from VMIs have high diagnostic efficiencies for differentiating low- and high-grade renal cell carcinoma. In our study, the radiomics model based on 50 keV and 150 kev images could help predict tumor invasiveness with an accuracy of 78.6% in the test set. Among the features included in the radiomics model, the firstorder_Median (media gray level intensity within the tumor) was closely associated with LUAD invasiveness with the highest estimate coefficient (1.99). We assumed that the difference in heterogeneity between the preinvasive-MIA and IA may be associated with their different density. Our results suggested that 50kev images combined with 150kev images can provide helpful information for predicting tumor invasiveness.

Several studies (38, 39) have analyzed radiomics for predicting the invasiveness of GGNs in LUAD. Zhang et al. (38) enrolled 65 patients with pGGNs and found that the radiomics model performed well in identifying invasive lung lesions with an AUC of 0.82. Weng et al. (39) enrolled 119 patients with part-solid nodules and found that the radiomics model had superior predictive performance compared to the radiographic model (AUC, 0.81 *vs.* 0.76). Previous studies mainly explored radiographic features and radiomics based on traditional CT. The current study, for the first time, used DECT-based radiomics to establish a predictive model (AUC, 0.96 and 0.87 in the training and test set, respectively) of LUAD invasiveness manifesting as GGNs. In addition to the different imaging techniques, the difference among the three studies might also be attributable to inclusion and grouping standards of the patients. Weng et al. included part-solid nodules, while Zhang et al. analyzed pure GGNs. In addition, Zhang et al. divided the nodules into preinvasive lesions (AAH and AIS) and invasive lesions (MIA and IA), whereas Weng et al. classified nodules into MIA and IA. Thus, larger samples and better designs are required in future studies to validate the present results. Despite the differences among various studies, these results still indicate that models from DECT can well estimate the invasiveness of LUAD. Furthermore, our radiomics model did not significantly outperform the clinical-DECT model in predictive ability. This may be because some of the imaging features we evaluated were probably contained in the radiomics features.

### Study limitations

There were several limitations to this study. First, this was a single-center retrospective study and the sample size was relatively small, which may constrain the generalizability of our findings. Second, we only included the CT features that have been reported and validated. The results may not reflect the complete CT morphological features of tumors. Third, the main purpose of the study was to identify tumor invasiveness of GGNs, and the density of nodules was not further subdivided. Finally, as the patients were examined from 2021 to 2022 with a limited follow-up time, the influence of IA and preinvasive-MIA on patient outcomes was not assessed.

## Conclusions

In summary, the DECT-based radiomics model showed satisfactory predictive performance in preoperatively differentiating IA and preinvasive-MIA. Further prospective multicenter studies are necessary to assess the utility of the model for clinical application.

## Data availability statement

The original contributions presented in the study are included in the article/**Supplementary Material**. Further inquiries can be directed to the corresponding authors.

## Ethics statement

The studies involving human participants were reviewed and approved by Ethics Committee of Wuhan Union Hospital. The ethics committee waived the requirement of written informed consent for participation.

## Author contributions

Conception and design: YZ, XH, XZ, HS. Administrative support: HS, XZ. Provision of study materials or patients: XJ, XC, XHZ. Collection and assembly of data: YZ, XH, KZ, HL. Data analysis and interpretation: CD, XC, XHZ. Manuscript writing: all authors. Final approval of manuscript: all authors.

## Supplementary material

Supplementary Figure 1 The Rad-score between groups in the training set **(A)** and test set **(B)**.

Supplementary Figure 2 Decision curves for the two models.

Supplementary Figure 3 Waterfall plot of the rad-score showing the predicted probabilities of the training **(A)** and test**(B)** sets.
