# Supplementary figures and images for: Dual-energy CT-based radiomics for predicting invasiveness of lung adenocarcinoma appearing as ground-glass nodules
Source: Front Oncol. 2023 Aug 10;13:1208758. doi: 10.3389/fonc.2023.1208758 (PMC10449576; doi:10.3389/fonc.2023.1208758)

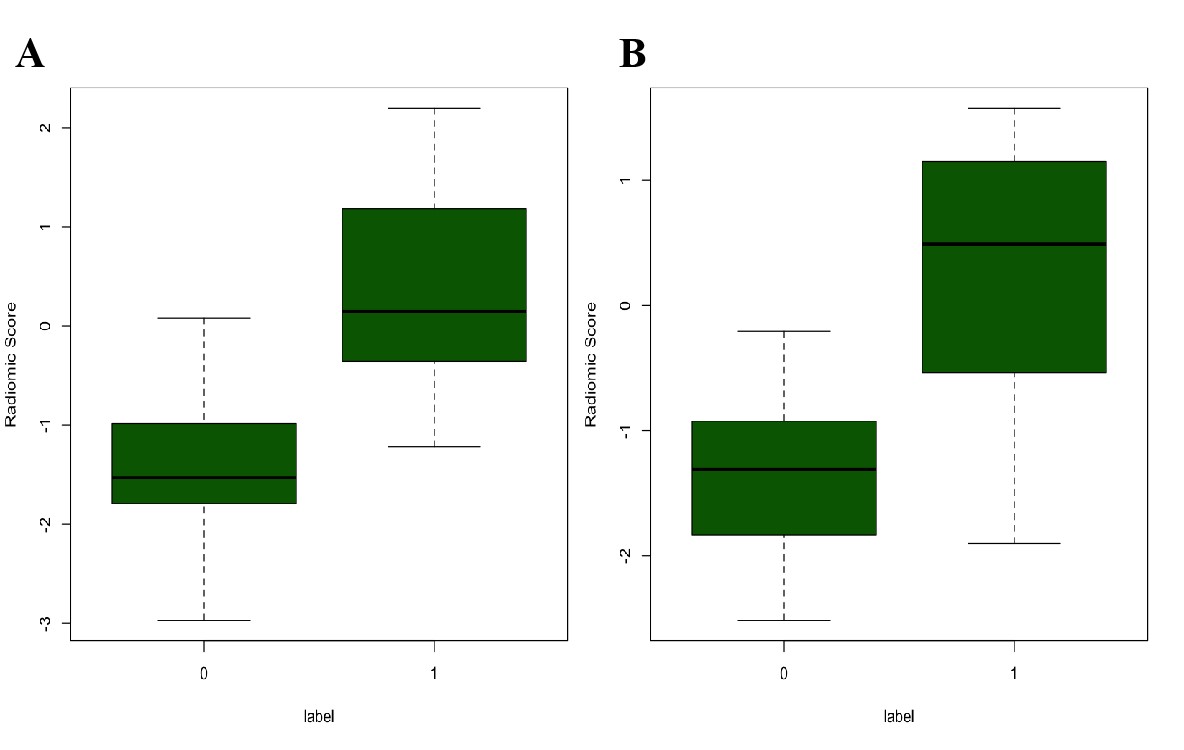

Supplement: Supplementary Figure 1 — The Rad-score between groups in the training set (A) and test set (B). [file Image_1.jpg]

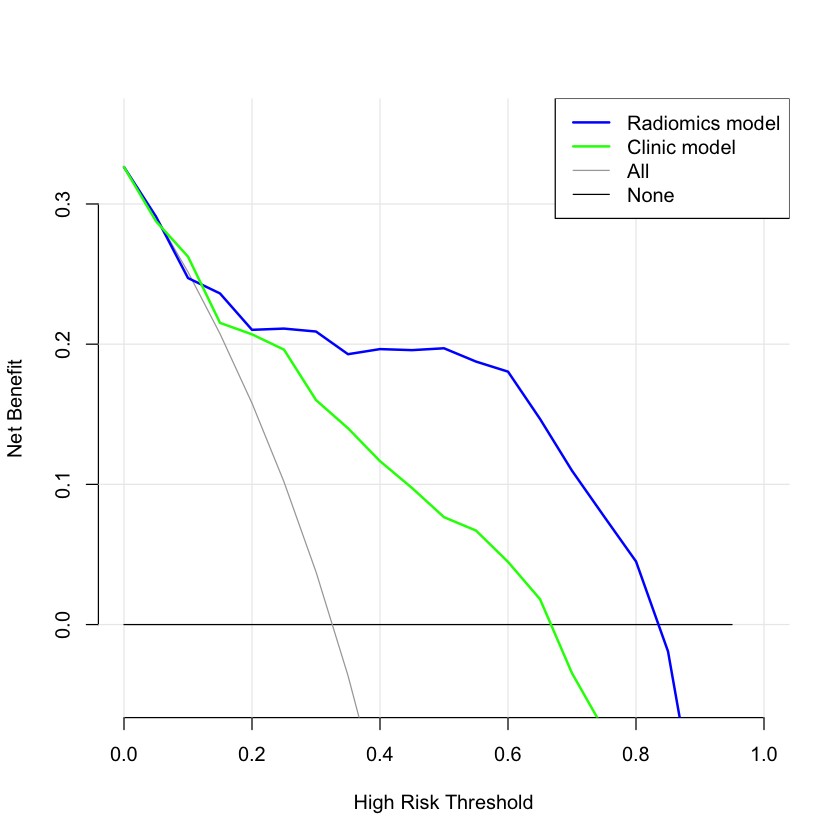

Supplement: Supplementary Figure 2 — Decision curves for the two models. [file Image_2.jpg]

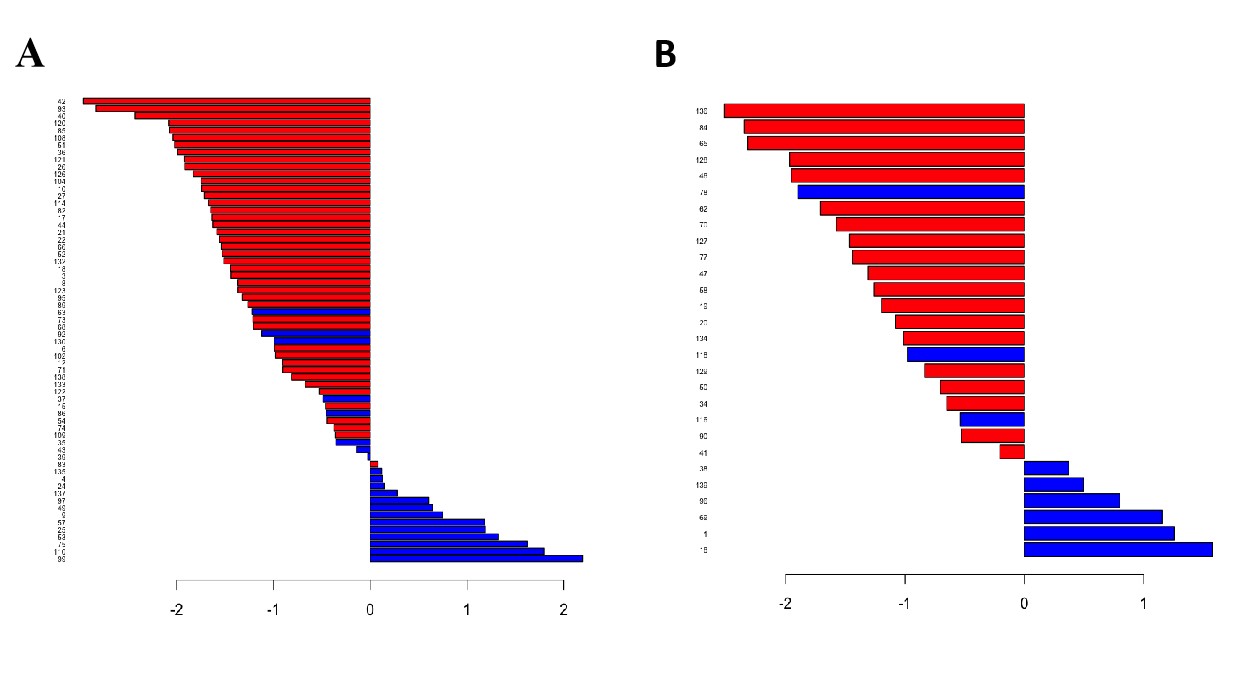

Supplement: Supplementary Figure 3 — Waterfall plot of the rad-score showing the predicted probabilities of the training (A) and test(B) sets. [file Image_3.jpg]
